# Modeling of variables in cellular infection reveals CXCL10 levels are regulated by human genetic variation and the *Chlamydia-*encoded CPAF protease

**DOI:** 10.1038/s41598-020-75129-y

**Published:** 2020-10-26

**Authors:** Benjamin H. Schott, Alejandro L. Antonia, Liuyang Wang, Kelly J. Pittman, Barbara S. Sixt, Alyson B. Barnes, Raphael H. Valdivia, Dennis C. Ko

**Affiliations:** 1 Department of Molecular Genetics and Microbiology, School of Medicine, Duke University, 0049 CARL Building Box 3053, 213 Research Drive, Durham, NC 27710 USA; 2 Duke University Program in Genetics and Genomics, Duke University, Durham, NC 27710 USA; 3 Division of Infectious Diseases, Department of Medicine, School of Medicine, Duke University, Durham, NC 27710 USA; 4 Present Address: Laboratory for Molecular Infection Medicine Sweden (MIMS), Umeå Centre for Microbial Research, Department of Molecular Biology, Umeå University, Umeå, Sweden

**Keywords:** Quantitative trait loci, Immunogenetics, Bacterial genes

## Abstract

Susceptibility to infectious diseases is determined by a complex interaction between host and pathogen. For infections with the obligate intracellular bacterium *Chlamydia trachomatis,* variation in immune activation and disease presentation are regulated by both host genetic diversity and pathogen immune evasion. Previously, we discovered a single nucleotide polymorphism (rs2869462) associated with absolute abundance of CXCL10, a pro-inflammatory T-cell chemokine. Here, we report that levels of CXCL10 change during *C. trachomatis* infection of cultured cells in a manner dependent on both host and pathogen. Linear modeling of cellular traits associated with CXCL10 levels identified a strong, negative correlation with bacterial burden, suggesting that *C. trachomatis* actively suppresses CXCL10. We identified the pathogen-encoded factor responsible for this suppression as the chlamydial protease- or proteasome-like activity factor, CPAF. Further, we applied our modeling approach to other host cytokines in response to *C. trachomatis* and found evidence that RANTES, another T-cell chemoattractant, is actively suppressed by *Chlamydia.* However, this observed suppression of RANTES is not mediated by CPAF. Overall, our results demonstrate that CPAF suppresses CXCL10 to evade the host cytokine response and that modeling of cellular infection parameters can reveal previously unrecognized facets of host–pathogen interactions.

## Introduction

Variation in how susceptible a host is to infectious diseases is in part shaped by the evolutionary pressure exerted on the human genome by pathogens. Classic examples of genetic variation underlying susceptibility to infection include the sickle cell trait (HbS; rs334) protecting against *Plasmodium falciparum*^1^ and the *ccr5* Δ32 allele (rs333) protecting against Human Immunodeficiency Virus^2^. With advances in high-throughput genotyping and next generation DNA sequencing technologies, the number and quality of genome-wide association studies has dramatically expanded, leading to the systematic identification of over 17,000 genome-wide significant associations with disease susceptibility^3^. However, in most cases, the link between genetic variants and specific cellular pathophysiology controlling susceptibility is poorly understood.

One approach to better understand the basis for this variation in pathophysiology is performing genome-wide association studies on in vitro infection and immune stimulation systems^4–9^. By using specific molecular and cellular phenotypes, association studies can be performed on intermediate phenotypes of infection that are likely governed by genetic variants with larger effect size compared to complex, multi-factorial organismal phenotypes. Additionally, by performing all infections with the same pathogen strain and dose, pathogen variation can be controlled. In pioneering work, Wurfel et al. 2008 identified variants in the *TLR1* region that were associated with cytokine levels following ex vivo stimulation of whole blood and outcomes in sepsis^10,11^. Similarly, we utilized a cellular GWAS platform called Hi-HOST (High-throughput in vitro Susceptibility Testing) to identify both cytokine quantitative trait loci (QTL)^9^ and single nucleotide polymorphisms (SNPs) that impacted the outcome of cellular infections such as pyroptosis^12,13^ and bacterial invasion^14^. Some of these same variants are associated with sepsis^12,15^, typhoid fever^14^, and other diseases^9,16^ in independent human cohorts. These studies demonstrate that analysis of cellular traits that contribute to complex disease phenotypes can reveal novel genetic loci associated with host response to pathogens.

However, even for molecular and cellular traits of infection, human genetic variation does not provide a complete explanation. A deeper understanding of inter-individual variation requires examining both host and pathogen factors. Previously we identified a SNP, rs2869462, as a QTL for levels of the chemokine CXCL10 (IP-10) after *Chlamydia trachomatis* infection in a cellular GWAS^9^. *C. trachomatis* is a leading cause of bacterial sexually transmitted disease globally^17^ that results in diverse diseases depending on both the host inflammatory response and bacterial factors^18^. Infection can remain asymptomatic, present as acute infection, or lead to pelvic inflammatory disease (PID) and infertility^19–21^. CXCL10 is produced after *C. trachomatis* infection in humans^22,23^ and mice^24–26^ and regulates the recruitment of CD4 + and CD8 + T-cells through the CXCR3 chemokine receptor. Because CXCR3 + cells can promote *C. trachomatis* clearance^27,28^ and immune-mediated pathology^29^, CXCL10 is a critical determinant of *C. trachomatis* disease. On the pathogen side, *C. trachomatis* has evolved myriad mechanisms to evade host immunity (reviewed in^30^) by interfering with key defense strategies such as nutrient restriction^31,32^, apoptosis^33–36^, reactive oxygen species (ROS) or nitric oxide (NO) production^37,38^, and T-cell function^39,40^. These immune evasion strategies have the potential to obscure acquired beneficial genetic variants for defense against *C. trachomatis* infection. Thus, observed levels of cytokines and other readouts of immune response result from the complex interplay of host variation and pathogen immune evasion.

In this study, we modeled the interindividual variation in cytokine responses to infer the presence of a *C. trachomatis* mechanism of chemokine suppression. We observed that rs2869462 genotype is a major contributor to levels of CXCL10 both at baseline and following infection but that the fold change of CXCL10 protein abundance after infection is not associated with the rs2869462 locus. In fact, fold change of CXCL10 after infection was not under the control of any large effect human genetic variant. Instead, linear modeling of the detailed infection parameters collected during the cellular GWAS of CXCL10 supported a model of bacterial suppression of chemokine levels. We identified the chlamydial protease-like activity factor (CPAF) as necessary for CXCL10 suppression during *C. trachomatis* infection. Finally, we repeated the linear modeling for additional cytokine traits and discovered *C. trachomatis* suppression of RANTES, but the mechanism of suppression was independent of CPAF. This work illustrates the power of cellular GWAS to reveal not only how human genetic differences impact disease outcomes but also provide unexpected contributions of pathogen-encoded factors underlying susceptibility to infectious diseases.

## Results

### rs2869462 associates with total, but not fold change, of CXCL10 protein abundance after infection with *Chlamydia trachomatis*

**Figure 1:**
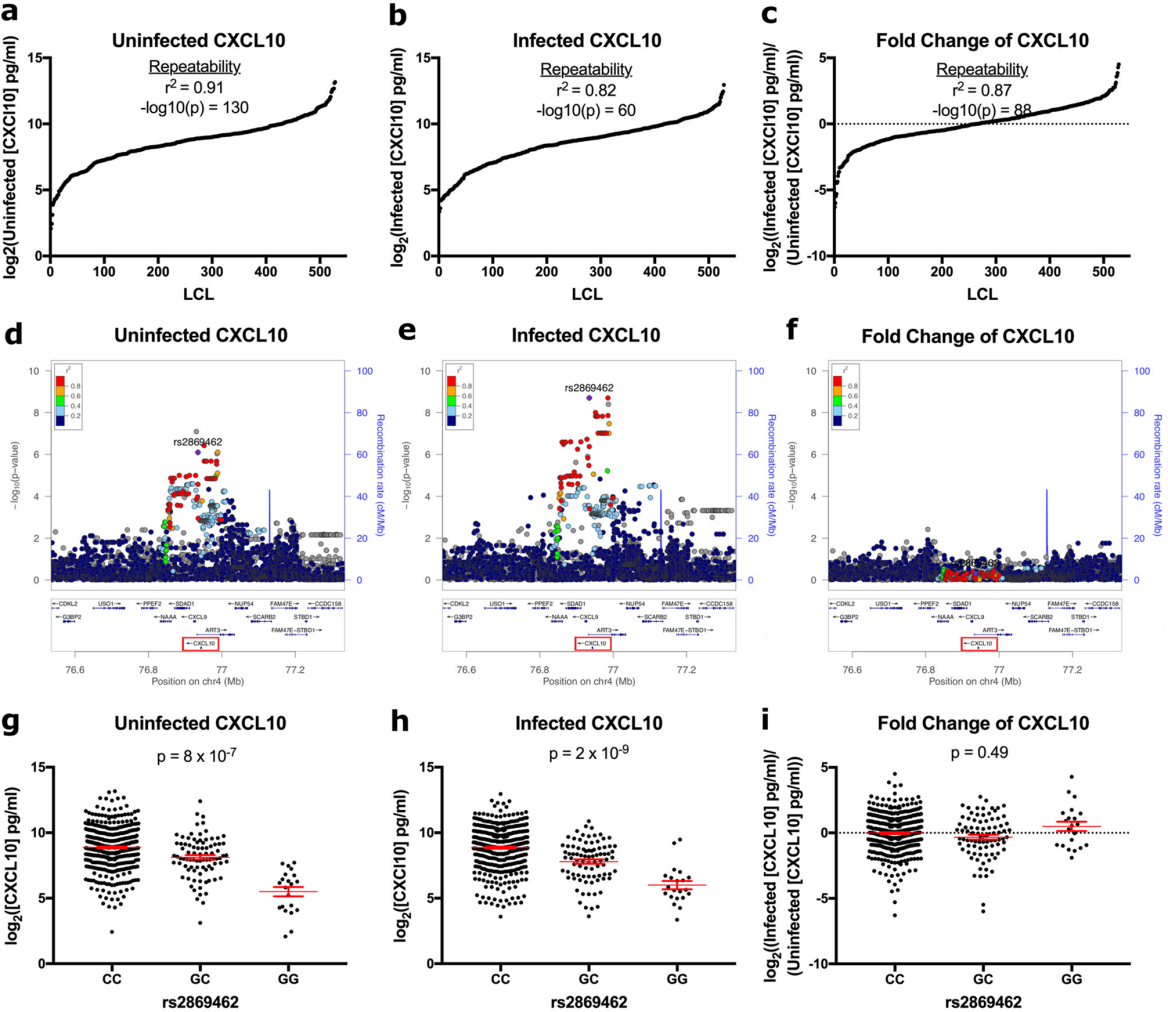
rs2869462 is associated with CXCL10 levels in uninfected and *C. trachomatis* infected cells but not fold change of CXCL10 in response to *C. trachomatis* infection. (**a**–**c**) CXCL10 levels in the uninfected state and after *C. trachomatis* infection of lymphoblastoid cell lines have high inter-individual variation and experimental repeatability. 528 LCLs from 4 populations in parent–offspring trios were infected with *C. trachomatis* LGV-L2 Rif^R^ pGFP::SW2. At 70 h post infection, cell culture supernatants were harvested and CXCL10 protein quantified by Luminex assay. The CXCL10 concentration was then used to calculate the following traits: (**a**) Uninfected CXCL10 (log_2_(uninfected [CXCL10] pg/ml)), (**b**) Infected CXCL10 (log_2_(infected [CXCL10] pg/ml)), and (**c**) Fold Change of CXCL10 (log_2_((Infected [CXCL10] pg/ml)/(Uninfected [CXCL10] pg/ml))). Repeatability (r^2^) shown is the estimated inter-individual variance calculated from duplicate experiments fitted with one-way ANOVA. (**d–f**) Uninfected and total infected CXCL10 traits have a peak of association 7.5 kb downstream of the *CXCL10* coding sequence on chromosome 4 that is absent from the fold change of CXCL10 trait. P-values were calculated as described previously^9^ using QFAM-parents in PLINK^42,43^ and plotted using LocusZoom^44^, with color-coding of r^2^ linkage disequilibrium values from 1000 Genomes^45^. The *CXCL10* gene is highlighted with a red box. Plots were generated separately for (**d**) uninfected CXCL10 levels, (**e**) infected CXCL10 levels, and (**f**) fold change of CXCL10. (**g**–**i**) rs2869462 is a quantitative trait locus for uninfected and total infected CXCL10, but not fold change of CXCL10 after *C. trachomatis* infection. 527 LCLs were grouped by rs2869462 genotype for the (**g**) uninfected, (**h**) infected, and (**i**) fold change of CXCL10 traits. P-values are from QFAM-parents in PLINK. While the minor G allele is strongly associated with lower levels of CXCL10 in uninfected and infected conditions, there is no association of rs2869462 with fold change of CXCL10.

We previously performed a cellular GWAS on CXCL10 levels in culture supernatants after *C. trachomatis* (LGV-L2 Rif^R^ pGFP::SW2) infection of 528 LCLs from 4 populations, ESN (Esan in Nigeria), GWD (Gambian in Western Divisions in the Gambia), KHV (Kinh in Ho Chi Minh City, Vietnam), IBS (Iberian in Spain), in parent–offspring trios. We identified a SNP (rs2869462) associated with CXCL10 protein levels after *C. trachomatis* infection (p = 2 × 10^–9^)^9^. Further analysis of the 528 LCLs revealed high inter-individual variation and experimental repeatability for three CXCL10 traits: Uninfected CXCL10 levels in supernatants of LCLs at baseline (log_2_(uninfected [CXCL10] pg/ml)), infected CXCL10 levels in supernatants of *C. trachomatis-*infected LCLs (log_2_(infected [CXCL10] pg/ml)), and fold change of CXCL10 levels, as the ratio of the infected and uninfected CXCL10 levels (log_2_((infected [CXCL10] pg/ml)/(uninfected [CXCL10] pg/ml))) (Fig. 1a–c; see Supplementary Table S1 for phenotype values). We hypothesized that rs2869462 is associated with increased expression of CXCL10 upon pathogen sensing by LCLs.

We observed that fold change of CXCL10 protein had very high repeatability for each individual LCL (p = 1 × 10^–88^; r^2^ = 0.87). However, there was variable levels of induction, with even suppression observed for half of the 528 LCLs (Fig. 1c). The rs2869462 SNP is 7.5 kilobases 3′ of the *CXCL10* coding sequence. This locus is associated with both uninfected (Fig. 1d; p = 8 × 10^–7^) and infected CXCL10 (Fig. 1e; p = 2 × 10^–9^) but not with the fold change of CXCL10 in response to *C. trachomatis* (Fig. 1f; p = 0.49). For uninfected CXCL10 (Fig. 1g) and total infected CXCL10 (Fig. 1h), the C allele was associated with higher protein levels. This quantitative trait locus was consistent with mRNA levels in the GTEx database, where the rs2869462 C allele significantly predicts higher *CXCL10* transcript in uninfected EBV-transformed lymphocytes (p = 4.9 × 10^–8^)^41^. This association is completely absent in the *C. trachomatis*-induced fold change of CXCL10 (Fig. 1i), suggesting that the change in CXCL10 after infection is not significantly explained by regulation through the rs2869462 locus.

### Fold change of CXCL10 in response to *C. trachomatis* infection is not explained by a human genetic variant of large effect

**Figure 2:**
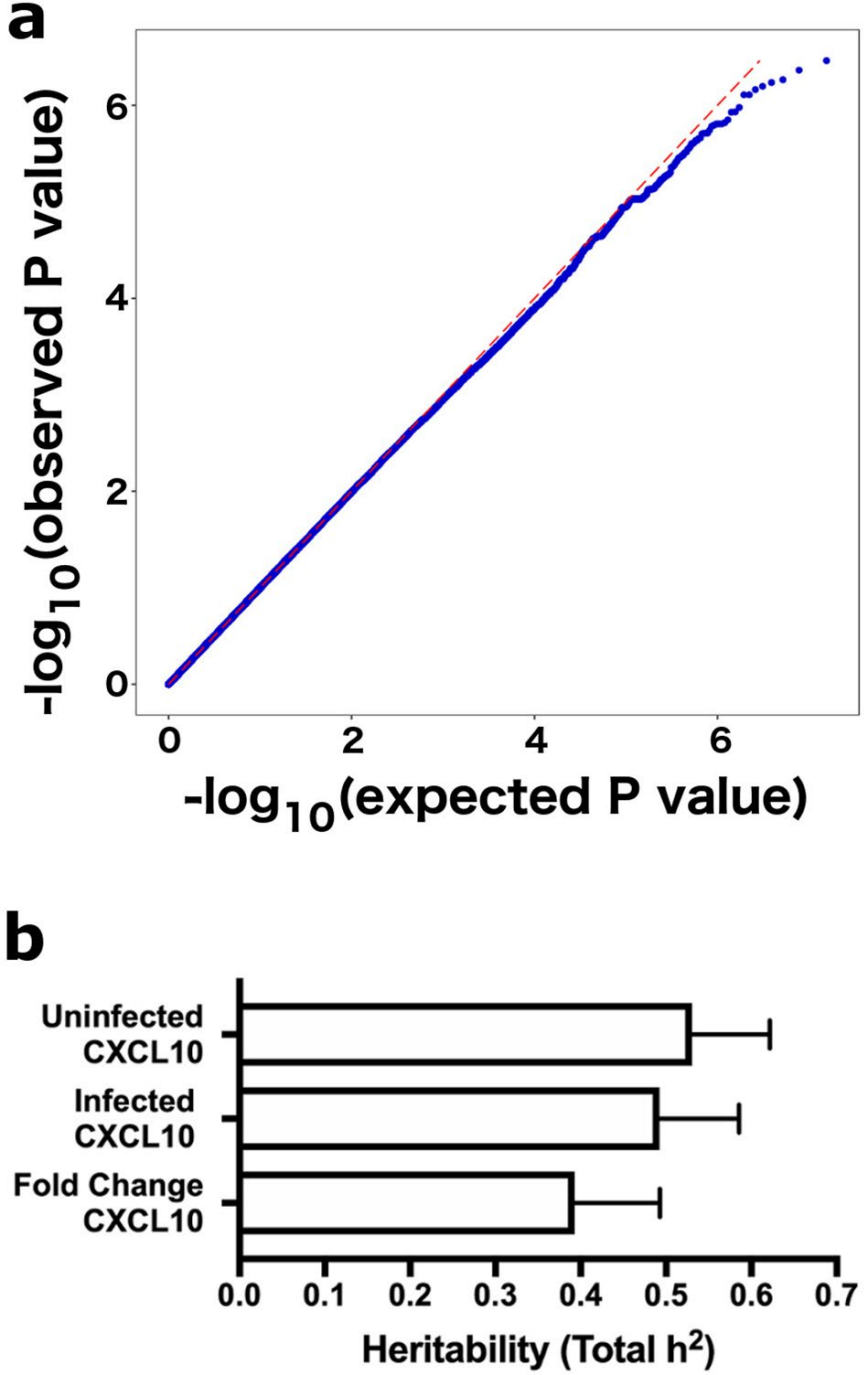
Fold change of CXCL10 in response to *C. trachomatis* infection is not strongly associated with a single genetic variant of large effect and has lower heritability than uninfected or infected CXCL10 levels. (**a**) Fold change of CXCL10 was not significantly associated with any single genetic variant. QQ plot generated with the quantile–quantile function in R displays − log(p-values) expected by chance for 8.8 million common SNPs (minor allele frequency > 5%) vs. observed − log(p-values). Note there is no evidence of any SNPs with p-values lower than expected by chance. (**b**) Fold change of CXCL10 has lower heritability than uninfected or infected CXCL10. Heritability and standard error were estimated using the variance components method of Zaitlen et al. 2013. This method calculates heritability explained by genetic relatedness and genome-wide SNP data.

Because rs2869462 was not associated with fold change of CXCL10 levels in response to *C. trachomatis* infection (Fig. 1), we tested whether the trait was under control of other human genetic variants. The quantile–quantile (QQ) plot of expected versus observed P values for all SNPs associated with the fold change of CXCL10 trait indicated that no single genetic variants are more strongly associated with this trait than predicted by a null distribution (Fig. 2a). To test whether the fold change of CXCL10 phenotype might be explained by polygenic contributions, we calculated the narrow-sense heritability (h^2^) of the three CXCL10 traits described above using the variance components method for related individuals from Zaitlen et al.^46^. While all three traits had significant h^2^, the fold change of CXCL10 trait (h^2^ = 0.39) was moderately less heritable than the infected CXCL10 trait (h^2^ = 0.49) (Fig. 2b). With no compelling basis for a host genetic variant of large effect regulating fold change of CXCL10, we next leveraged additional data generated during the cellular phenotyping of infected cells to identify the factors driving the variation in the CXCL10 response.

### Linear modeling reveals a strong contribution of *Chlamydia* burden to fold change of CXCL10

**Figure 3:**
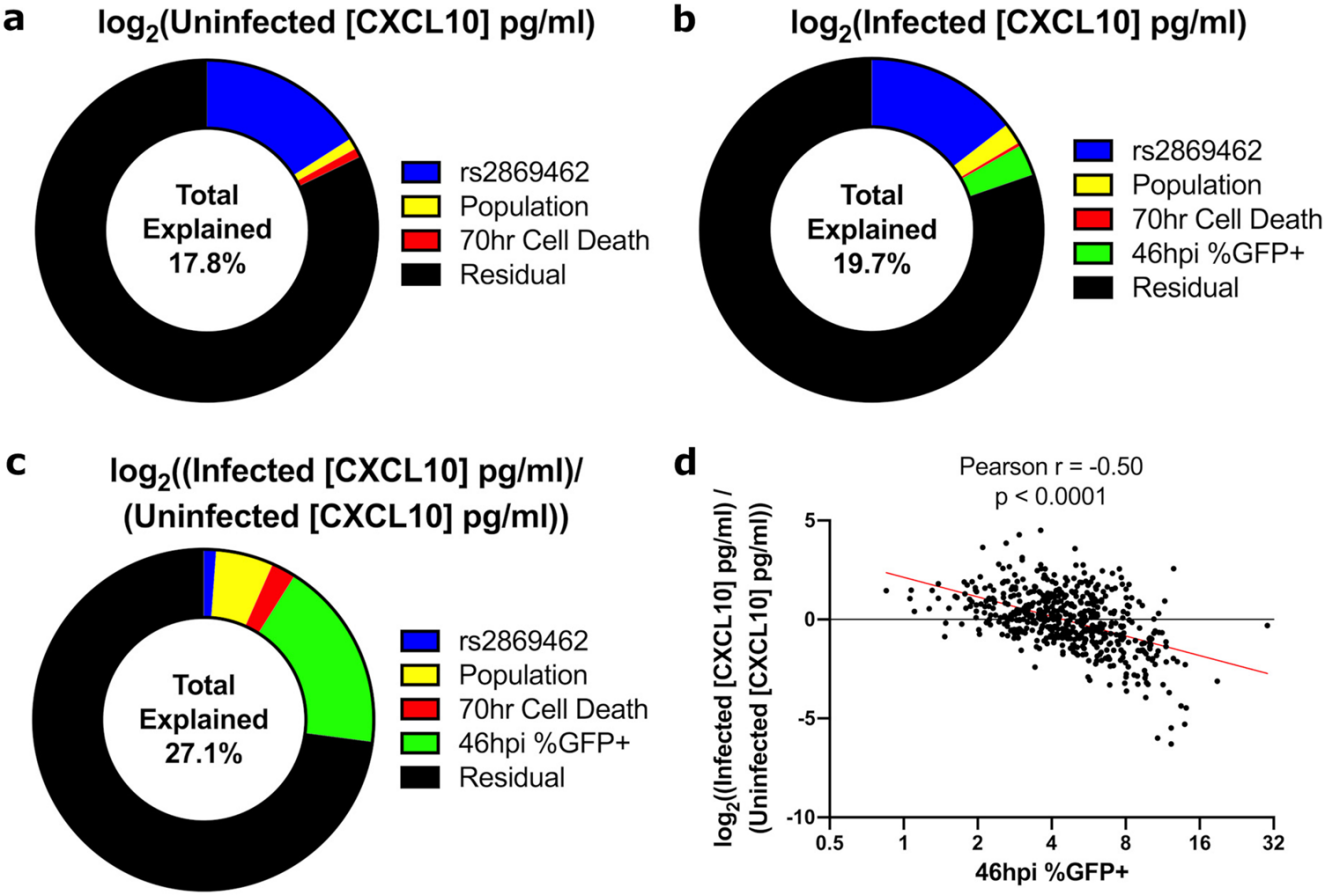
Modeling CXCL10 abundance phenotypes as a function of host genetic and cellular GWAS factors reveals *C. trachomatis* burden strongly determines fold change of CXCL10 after infection. The largest explanatory variable for uninfected and infected CXCL10 is host rs2869462 genotype, but *C. trachomatis* burden explains the largest fraction of variation in fold change of CXCL10. (**a**) Linear modeling of uninfected CXCL10 protein concentration in cell culture supernatants at 70 h (log_2_(uninfected [CXCL10] pg/ml)) as a function of host genotype at rs2869462, population, and 70 hr cell death. (**b**,**c**) Linear modeling of CXCL10 protein concentration in cell culture supernatants at 70 h post infection (log2(infected [CXCL10] pg/ml)) (**b**) and fold change of CXCL10 (log2((Infected [CXCL10] pg/ml)/(Uninfected [CXCL10] pg/ml))) (**c**) as a function of rs2869462, population, 70hpi cell death, and 46hpi %infected cells. For fold change of CXCL10, the effect of rs2869462 is reduced, while the effect of bacterial burden is increased. (**d**) Scatter plot of fold change of CXCL10 (log2((Infected [CXCL10] pg/ml)/(Uninfected [CXCL10] pg/ml))) vs. 46hpi % infected cells showing a highly significant negative correlation (Pearson correlation p < 0.0001; r = − 0.50).

We used a multiple linear modeling approach to quantify the contributions of cellular infection variables measured during Hi-HOST cellular GWAS of *C. trachomatis*. Each CXCL10 phenotype (uninfected, infected, and fold change) was expressed as the linear combination of up to four explanatory variables: rs2869462, population, cell death, and *C. trachomatis* burden. First, we considered the effect of rs2869462 because it is a SNP of large effect associated with protein levels with and without infection (Fig. 1). Second, we considered the LCL population (KHV, GWD, ESN, or IBS) as a measure of the influence of population-specific effects on CXCL10. Third, we considered cell death at 70 h post infection to account for decreased CXCL10 due to a lower number of viable cells. Fourth, we considered *C. trachomatis* (LGV-L2 Rif^R^ pGFP::SW2) burden defined as the percent of LCLs measured as GFP positive at 46 h post infection. For uninfected CXCL10, where bacterial burden could not be included as a variable, the genotype at rs2869462 was responsible for 15.9% of the variation, the population variable explained 1.0% of the residual variation, and cell death explained 0.9% of the subsequent residual variation (Fig. 3a). For infected CXCL10, rs2869462 explained 14.5% of the total variation, population explained 2% of the residual variation, cell death explained 0.3% of the subsequent residual variation, and bacterial burden explained 2.9% of the final residual variation (Fig. 3b). Finally, for fold change of CXCL10, rs2869462 explained 1.2% of the total variation, population explained 5.5% of the residual variation, and cell death explained 2.3% of the subsequent residual variation. *C. trachomatis* burden explained 18.1% of the final residual variation, indicating that variation in *Chlamydia* levels is a primary driver of this phenotype (Fig. 3c).

Given that a primary driver of fold change of CXCL10 was *C. trachomatis* burden, we hypothesized that increased bacterial sensing by innate immune receptors in highly infected cells was leading to heightened responses. Surprisingly, we observed a negative correlation between *C. trachomatis* burden and CXCL10 levels (Fig. 3d, Pearson r = − 0.50). Therefore, we inferred the presence of a *Chlamydia*-produced factor present in higher abundance in more heavily infected cells acting to suppress CXCL10. This inference from modeling cellular infection is consistent with previous observations that *C. trachomatis* suppresses levels of chemokines including CXCL10 in A2EN endocervical epithelial cells by unknown mechanisms^47^.

### *Chlamydia* suppression of CXCL10 is mediated by the chlamydial protease-like activity factor (CPAF)

**Figure 4:**
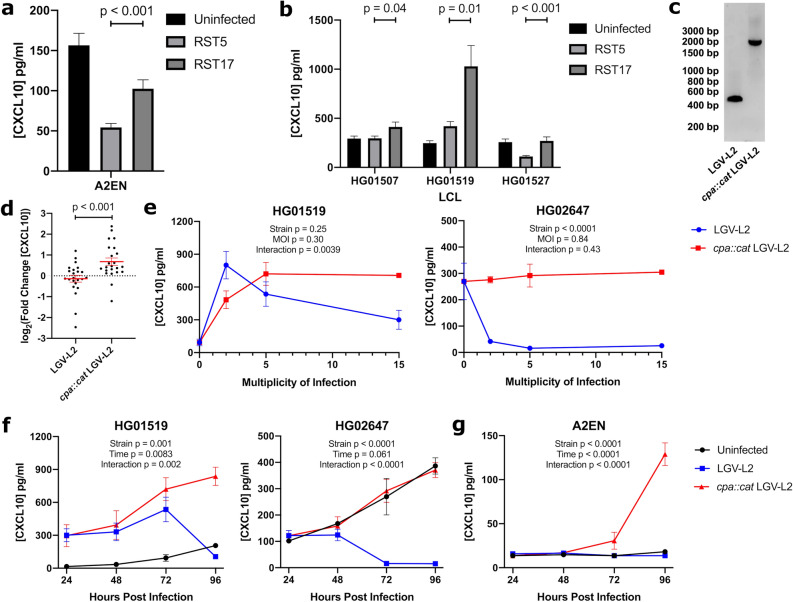
Suppression of CXCL10 following *C. trachomatis* infection is mediated by the pathogen-encoded protease, CPAF. (**a**,**b**) Chemically generated CPAF mutant increases levels of CXCL10 compared to WT *C. trachomatis* infection. A2ENs (**a**) and three LCLs (**b**), HG01507, HG01519, and HG01527 were infected with a CPAF^+^ (RST5) or CPAF^−^ (RST17) LGV-L2 strain of *C. trachomatis* at MOI 5. At 70 h post infection, cell culture supernatants were harvested and assayed for CXCL10 concentration by ELISA. Data are from 12 biological replicates per condition across 2 experiments. P-values are from unpaired t-tests. (**c**) DNA gel confirming ~ 1.5 kb TargeTron-mediated insertion in *cpa*::*cat* LGV-L2 strain. (**d**) TargeTron-generated CPAF mutant increases fold change of CXCL10 during *C. trachomatis* infection. 23 LCLs were infected at MOI 5 with *cpa*::*cat* LGV-L2 or WT parental LGV-L2. Cell culture supernatants were collected at 70 h post infection and assayed by ELISA. Data is represented as log_2_((infected [CXCL10] pg/ml)/(uninfected [CXCL10] pg/ml)). Each point is the mean from 3 biological replicates of each LCL across 3 experiments. P-values are from paired t-test. (**e**) Dose–response of infection with *cpa*::*cat* LGV-L2 or WT parental LGV-L2 in HG01519 and HG02647. Cell culture supernatants were collected at 72 h post infection and assayed by ELISA. Increasing MOI from 2 to 15 results in greater CXCL10 suppression in the WT strains while *cpa*::*cat* LGV-L2 strain demonstrates increasing CXCL10 with increasing MOI. P-values are from two-way ANOVA comparing *cpa*::*cat* LGV-L2 and WT LGV-L2 infected values. Note that for HG02647, the effect of CPAF is apparent at all MOIs resulting in a significant p-value for the effect of strain (p < 0.0001). For HG01519, the effect of CPAF is only apparent at the highest MOI, resulting in a significant p-value (p = 0.0039) for the interaction of strain and MOI. (**f**,**g**) Time-course of infection with *cpa*::*cat* LGV-L2 or WT parental LGV-L2 at MOI 5 in HG01519 and HG02647 (**f**) or A2EN (**g**). Cell culture supernatants were collected at 24, 48, 72, and 96 h post infection and assayed by ELISA. Similar levels of CXCL10 were detected at 24 and 48 h and increasing levels of CPAF-dependent suppression is observed from 72 to 96 h. P-values are from two-way ANOVA comparing *cpa*::*cat* LGV-L2 and WT LGV-L2 infected values. Data for E, F and G are from 4 biological replicates per condition from 2 experiments.

*C. trachomatis* encodes multiple virulence factors that can manipulate host cellular processes (reviewed by^30^). We hypothesized that chlamydial protease-like activity factor (CPAF) could contribute to CXCL10 suppression due to its reported role in subverting host immunity by cleaving substrates such as intermediate filaments to promote intracellular survival^48,49^ and interfering with NFκB signaling to alter the cytokine response^50^. A chemically derived CPAF^-^ strain of LGV-L2 (RST17) and a CPAF^+^ co-isogenic strain (RST5)^49,51^ allowed us to test whether CPAF is required for CXCL10 suppression in the cervical human epithelial cell line A2EN. At 70 h post infection, supernatants from cells infected with RST17 (CPAF^-^) had 48 pg/ml (88%) more CXCL10 than those infected with RST5 (CPAF^+^) (Fig. 4a; p < 0.001). A similar suppression was observed in LCLs, where infection with RST17 (CPAF^-^) led to 295 pg/ml (107%) more CXCL10 on average for three LCLs compared to the RST5 (CPAF^+^) infection (Fig. 4b). To confirm that CPAF was responsible for the in vitro suppression of CXCL10, we generated a second CPAF deficient strain of *C. trachomatis* L2 by targeted insertional mutagenesis (Fig. 4c). Across a panel of 23 LCLs, cells infected with the *cpa*::*cat* LGV-L2 mutant showed an increased fold change of CXCL10 relative to cells infected with the isogenic WT LGV-L2 strain (Fig. 4d; p < 0.001). Together, these two independently derived CPAF-deficient isolates demonstrate that CPAF is required for *C. trachomatis* suppression of CXCL10.

Dose–response and timecourse experiments further elucidated CPAF-dependent suppression as a late response during infection. Dose–response curves demonstrated that while *cpa*::*cat* LGV-L2 infection of LCLs have increasing CXCL10 induction with higher MOI, WT LGV-L2 showed greater suppression at higher MOI (Fig. 4e). This suppression appeared to occur only late during infection, beginning at 72 h and being nearly complete by 96 h (Fig. 4f), consistent with release of CPAF during host-cell lysis, though the mechanism of suppression of CXCL10 requires further biochemical investigation. Notably, much greater suppression is seen in HG02647 at lower MOIs and earlier time compared to HG01519, which is consistent with the large variation in CXCL10 fold change observed across all 528 LCLs at 70 h (see Fig. 1c). This time-dependent suppression of CPAF was also observed in A2EN cells, where the largest difference occurred at 96 h (Fig. 4g).

### *C. trachomatis* suppresses RANTES through a CPAF-independent mechanism

**Figure 5:**
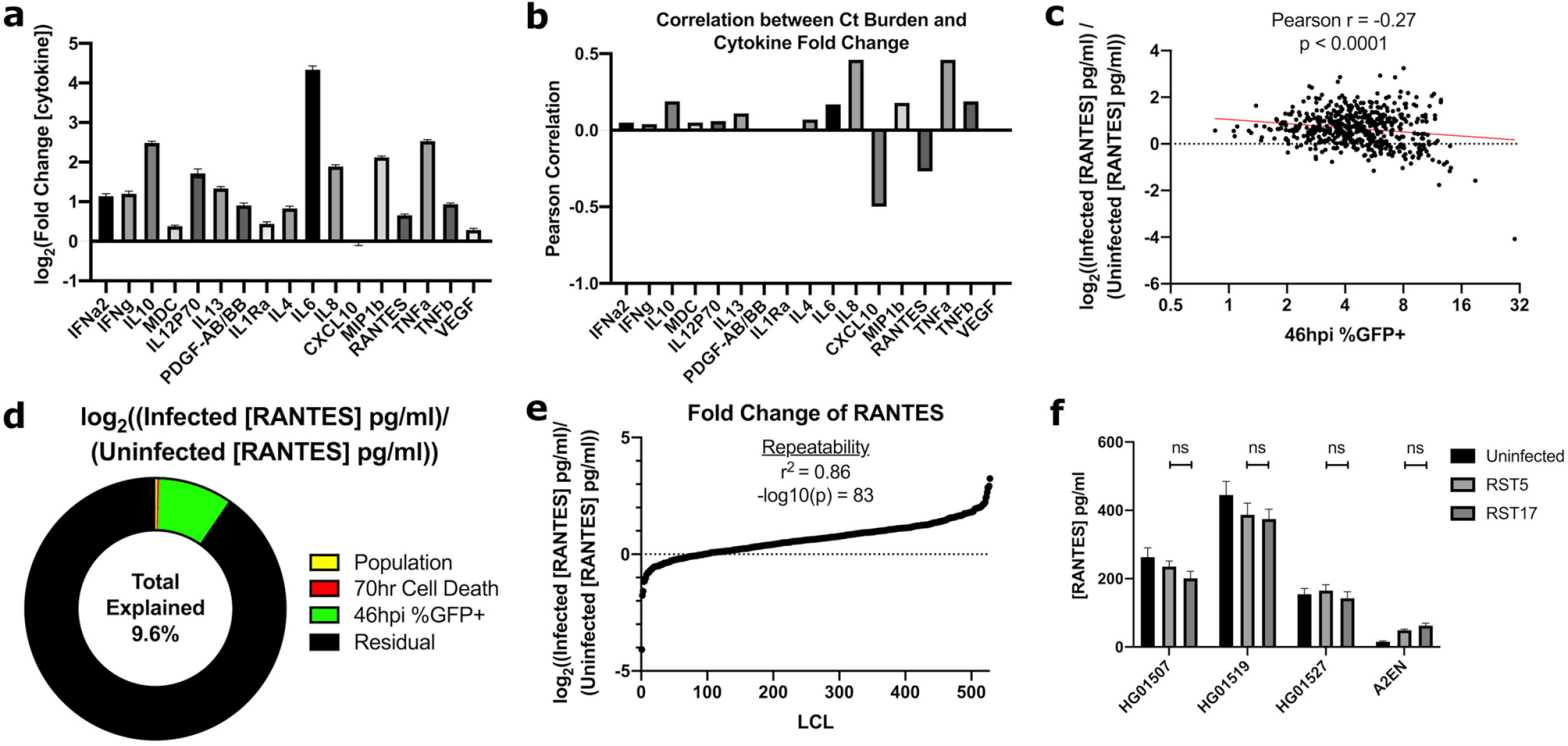
Modeling fold change of cytokine concentration as a function of bacterial burden reveals CPAF-independent suppression of RANTES. (**a**) All measured cytokines except CXCL10 demonstrate an average increase in concentration following *C. trachomatis* infection. 70 h-post-infection cell culture supernatants from 528 LCLs assayed by Luminex for 17 cytokines (log_2_(infected [cytokine]/uninfected [cytokine]). (**b**) CXCL10 and RANTES demonstrate a negative correlation with bacterial burden. Pearson correlation between 46 h-post-infection % infected cells and log2(infected [cytokine]/uninfected [cytokine]) for 17 cytokines, correlation is calculated over 528 LCLs. (**c**) Scatter plot of fold change of RANTES (log_2_((Infected [RANTES] pg/ml)/(Uninfected [RANTES] pg/ml))) vs. 46hpi % infected cells showing a highly significant negative correlation (Pearson correlation p < 0.0001; r = -0.27). (**d**) Linear modeling of fold change of RANTES (log2((Infected [RANTES] pg/ml)/(Uninfected [RANTES] pg/ml))) as a function of population, 70hpi cell death, and 46hpi %infected cells showing that *C. trachomatis* burden has a larger effect on fold change of RANTES than population or cell death. 528 lymphoblastoid cell lines from 4 populations in parent–offspring trios were infected with *C. trachomatis* LGV-L2 RifR pGFP::SW2. At 70 h post infection, cell culture supernatants were harvested and RANTES protein quantified by Luminex. (**e**) Distribution of suppression and induction of RANTES after *C. trachomatis* infection show most LCLs have an overall increase in RANTES while 94 LCLs demonstrate a decrease. (**f**) CPAF does not regulate concentration of RANTES. Three LCLs, HG01507, HG01519, and HG01527 and A2ENs were infected with CPAF + (RST5) or CPAF- (RST17) LGV-L2 strain of *C. trachomatis*. At 70 h post infection cell culture supernatants were harvested and assayed for RANTES concentration by ELISA. Data are from 12 biological replicates across 2 experiments. P-values are from unpaired t-tests.

We next tested if our modeling-based approach to infer pathogen-mediated suppression of host cytokines could be extended to a panel of 17 additional cytokines measured by Luminex after *C. trachomatis* infection during our previous Hi-HOST screen^9^. Only CXCL10 had, on average, a negative fold change after infection of 528 LCLs, and that difference was very close to no change (Fig. 5a). However, when considering correlation with bacterial burden (as we did for CXCL10 in Fig. 3d), we found that most cytokines (15/17) had a positive correlation with burden while CXCL10 and RANTES fold change had a negative correlation with burden (Fig. 5b). The negative correlation between burden and RANTES fold change was highly significant (Fig. 5c, Pearson r = − 0.27, p < 0.0001). To further explore the cellular infection variables affecting fold change of RANTES, we performed multiple linear modeling of this phenotype as a function of population, 70 h cell death and 46 h % infected cells. Population explained 0.2% of the variation, 70 h cell death explained 0.3% of the residual variation, and *C. trachomatis* burden explained 9.1% of the final residual variation (Fig. 5d). Across 528 LCLs, 434 LCLs demonstrated increased RANTES levels following infection while 94 LCLs demonstrated a decrease (Fig. 5e). Thus, like CXCL10, there appears to be simultaneous host induction of RANTES and suppression by *C. trachomatis.* Interestingly, RANTES, like CXCL10, is a T-cell chemoattractant that augments the host inflammatory response to *C. trachomatis* infection and decreases acute bacterial burden but can increase the risk of inflammatory sequelae such as infertility^26,52,53^.

As both CXCL10 and RANTES promote T-cell recruitment to the infection site, we hypothesized that CPAF may suppress RANTES levels similarly to CXCL10. To test this, we measured RANTES levels after infection of LCLs with *C. trachomatis* L2 RST5 (CPAF^+^) or RST17 (CPAF^-^); however, there was no CPAF dependent reduction in RANTES (Fig. 5f). Therefore, it appears that the potential suppression of RANTES is not mediated by CPAF, and this result highlights the specificity of CPAF-mediated suppression of CXCL10. Ultimately, careful modeling of cellular GWAS traits has thus revealed that there are multiple host pathways suppressed by *C. trachomatis.*

## Discussion

We described a detailed analysis of inter-individual variation of cellular responses of LCLs to *C. trachomatis* infection to predict the presence of a pathogen-encoded factor capable of suppressing the pro-inflammatory chemokine, CXCL10. While fold change of CXCL10 levels after *C. trachomatis* infection demonstrated a high degree of phenotypic variation and repeatability, it is not under the control of a single genetic locus of large effect. Multiple linear modeling of cellular infection variables identified *C. trachomatis* burden as the largest known contributing variable. This phenotype was paradoxically negatively correlated with fold change of CXCL10. This suggested that *C. trachomatis* could be producing a factor in higher abundance in more highly infected cell populations to suppress CXCL10. We identified a *Chlamydia*-encoded protease, CPAF, as necessary for this suppression using two independently generated mutant strains. Characterizing the physiological effects of CPAF has been complicated in early studies by the lack of tools for genetic ablation of CPAF and by the unawareness of incomplete inactivation of protease activity during preparation of cell lysates^49,54^. Notably, we observed CPAF-mediated suppression of CXCL10 levels with both chemical and insertion mutants, and our method of measuring CXCL10 in the supernatants does not involve cell lysis. Still, the molecular mechanism of CPAF-dependent suppression of CXCL10 remains to be elucidated. The kinetics of CXCL10 suppression are delayed during *C. trachomatis* infection of cultured cells, making CXCL10 suppression through an effect on cellular signaling less likely and consistent with release of CPAF and direct cleavage. However, CPAF has also been reported to facilitate lysis of host cells^55^, so our data are also consistent with CPAF mediating release of a host or chlamydial factor that mediates suppression. Further biochemical characterization of CXCL10 suppression will be required to resolve the mechanism of CXCL10 suppression.

We demonstrated the generalizability of these methods, and the specificity of CPAF suppression of CXCL10, by modeling the cellular traits contributing to 17 other cytokines. This approach revealed suppression of a second cytokine, RANTES, by a CPAF-independent mechanism. Thus, our method of leveraging natural diversity in *C. trachomatis* levels between diverse LCLs allowed us to infer the presence of pathogen-encoded factors affecting the induction of two crucial T-cell chemoattractants, CXCL10 and RANTES. While RANTES suppression may not be mediated by CPAF, *Chlamydia* species encode more than two dozen proteins with putative proteolytic roles^56^. Ultimately, we were able to uncover a layer of bacterial regulation of cytokine responses despite only modeling variables that explain up to 27% of cytokine variation. It is possible that more detailed insights into mechanisms of cytokine regulation by host and pathogen could emerge with cellular GWAS studies that include additional variables such as bacterial genetic variation, environmental confounders, or co-infection with other pathogens.

We previously identified a mechanism of CXCL10 suppression in the context of *Leishmania* infection mediated by the *Leishmania*-encoded matrix metalloprotease, GP63^57^. We found that this mechanism of immune evasion is beneficial to the parasite as it inhibits recruitment of anti-parasitic CXCR3 + T-cells. The observation that multiple pathogenic microorganisms have evolved such a mechanism is evidence that intracellular pathogens can gain an advantage by suppressing the human chemokine response.

Our findings may have implications for both infectious and autoimmune diseases. CXCL10 is a pro-inflammatory chemokine involved primarily in the recruitment of T cells to the site of infection, allowing for amplification of an inflammatory, antimicrobial immune response^58^. Previous work by Maxion and Kelly demonstrated strong induction of CXCL10 at 3 days post infection with *C. trachomatis* in the upper genital tracts of mice, followed by decreasing CXCL10 levels thereafter^25^. Our finding that *C. trachomatis* has evolved a mechanism to actively suppress CXCL10 supports an anti-bacterial role for CXCL10 during *C. trachomatis* infection, where the bacterium gains an advantage by suppressing the chemokine. In other infectious disease contexts, increased chemokine expression is also host-beneficial, leading to increased resistance to *Legionella pneumophila*^59^ and *Toxoplasma gondii*^60^. However, it has been demonstrated that IFNAR^-/-^ mice clear *C. trachomatis* infection quicker and develop less oviduct pathology relative to WT mice. Notably, CXCL10 is reduced in genital secretions of IFNAR^-/-^ mice relative to WT mice^61^. Moreover, CXCL10 and related chemokines have recently been found to promote immune-mediated pathology during *C. trachomatis* infection in mice^29^. Similarly, a recent study of women with *C. trachomatis* infection found elevated levels of CXCL10 in cervical secretions to be associated with endometrial chlamydial infection^23^.

Further, we have shown previously that SNPs affecting expression of CXCL10 are also associated with risk of inflammatory bowel disease (IBD), with the direction of affect indicating that the rs2869462 risk allele for IBD (C allele) is associated with higher levels of CXCL10^9^. Increased expression of CXCL10 has also been measured from colonic biopsies from patients with IBD^62^. Accordingly, the host must balance the expression of CXCL10, as too little signaling predisposes one to infectious disease, but too much signaling predisposes individuals to immune-mediated damage and autoimmunity. This genetic balancing act is complicated by pathogen mechanisms, such as those mediated by CPAF, that subvert immune responses during infection. Our results highlight the utility of integrating the multiple variables measured during cellular GWAS to maximize our understanding of host and pathogen determinants of infection and immune response.

## Materials and methods

### Cell lines

1000 Genomes LCLs used in this study were described previously^9^*.* Briefly*,* 1000 Genomes LCLs (528, all trios) from 4 populations (Kinh in Ho Chi Minh City, Vietnam, KHV; Iberian in Spain, IBS; Gambian in Western Divisions in The Gambia; GWD; Esan in Nigeria; ESN) were purchased from the Coriell Institute. LCLs were grown in RPMI 1640 media (Invitrogen) supplemented with 10% fetal bovine serum (FBS, Thermo 10438026), 2 mM l-glutamine (Thermo 25030081), 100U/ml penicillin–streptomycin (Thermo 15140122). Human Endocervical Epithelial Cell Line, A2ENs obtained from Raphael Valdivia, were grown in defined KSFM media (Thermo; 10744019) supplemented with 10% FBS. All human cell lines were grown at 37 °C with 5% CO2.

### *Chlamydia trachomatis* strains and infections

*C. trachomatis* LGV-L2 Rif^R^ pGFP::SW2, WT *C. trachomatis* LGV-L2, RST5 LGV-L2, RST17 LGV-L2, or Targetron-mediated CPAF mutant LGV-L2 was grown and purified as previously described^49,51,63^. Each preparation was titered uniformly by counting inclusion forming units on monolayers of Vero cells. *C. trachomatis* was diluted in RPMI with 10% FBS and added at MOI 5 in 100 µl in 96-well plates, mixed via pipetting, and centrifuged onto cells at 1500×*g* for 30 min at 4 °C. At 27, 46, and 70 h, cells were mixed and 25 µl was used for flow cytometry quantification of *C. trachomatis* burden and cell death. At 70 h, 25 µl of cell culture supernatant was measured by custom Luminex assay for 17 human cytokines (Millipore), or 50 µl of cell culture supernatant was measured by ELISA for [CXCL10] or [RANTES] (R&D).

### TargeTron-mediated generation of CPAF-deficient *C. trachomatis* LGV-L2

The gene encoding CPAF (*cpa*, CTL0233) in *C. trachomatis* LGV-L2 was disrupted using the TargeTron approach^64^. The CTL0233 gene sequence was scanned for potential target insertion sites using the TargeTron™ algorithm (Sigma-Aldrich). The predicted target site closest to the start codon of CTL0233 (between nucleotides 35 and 36) was chosen. The primers IBS-CTL0233 (5′- AAAAAAGCTTATAATTATCCTTAATTACCGCTTACGTGCGCCCAGATAGGGTG-3′), EBS1d-CTL0233 (5′- CAGATTGTACAAATGTGGTGATAACAGATAAGTCGCTTACCTTAACTTACCTTTCTTTGT-3′), EBS2-CTL0233 (5′- TGAACGCAAGTTTCTAATTTCGGTTGTAATCCGATAGAGGAAAGTGTCT-3′), and EBS universal (Sigma-Aldrich) were used to retarget vector pDFTT3-CAT^65^ for disruption of CTL0233. The resulting vector pDFTT3-CAT-CTL0233 was transformed into *C. trachomatis* LGV-L2 using the CaCl_2_-mediated approach^66^, with modifications described recently^67^. Transformed bacteria were selected in presence of 0.5 µg/ml chloramphenicol (Sigma-Aldrich), first added at 14 h post infection (hpi). Bacteria were then plaque-purified in presence of 1 µg/ml chloramphenicol. Intron insertion at correct target sites was verified by PCR using primers CTL0233_F/R (5′-GGCTGGTATGTCAAACACGC-3′ and 5′-CGCAAAGAAAGTTACTCCAGCG-3′) and by sequencing of the resulting PCR product (Eton Bioscience). The mutant strain is referred to as *cpa*::*cat* LGV-L2.

### High-throughput human in-vitro susceptibility testing (Hi-HOST)

Human variation in cytokine and chemokine production was measured by Hi-HOST as previously described^9^. Briefly, lymphoblastoid cell lines (LCLs) derived from four populations (ESN, IBS, KHV, and GWD) were obtained from Corriell and allowed to recover for 8 days in culture media. For screening assays, LCLs were counted using a Guava Easycyte Plus flow cytometer (Millipore), washed with RPMI 1% FBS, and plated at 40,000 cells per well in 100 μl RPMI 10% FBS in 96 well plates. LCLs were either uninfected or infected with *C. trachomatis* LGV-L2 Rif^R^ pGFP::SW2 at an MOI of 5. At the indicated time points, cells were run through a Guava EasyCyte Plus flow cytometer (Millipore) to assay cellular phenotypes of cell death by 7AAD + staining and bacterial burden by the *C. trachomatis* GFP reporter. The remaining cell cultures were centrifuged at 200×*g* for 5 min and the supernatants stored at − 80 °C for downstream cytokine quantification.

### Cytokine quantification

Cytokine concentration was measured by Luminex or enzyme-linked immunosorbent assay (ELISA) as indicated in the text. For genome-wide association studies on cytokine response, a custom Luminex assay was obtained from Millipore to detect 17 cytokines and chemokines: IFNα2, IFNγ, IL10, MDC, IL12p70, IL13, PDGF-AB/BB, IL1Rα, IL4, IL6, IL8, CXCL10, MIP1β, RANTES, TNFα, TNFβ, and VEGF. For follow up studies confirming suppression, chemokines were quantified by ELISA for CXCL10 (R&D; DY266) or RANTES (R&D; DY278).

### Genome-wide association and heritability estimation

Population genetic analyses were performed as described previously^9^ for the following 3 CXCL10 phenotypes: uninfected CXCL10, infected CXCL10, and fold change of CXCL10. For uninfected and total infected CXCL10, all analysis was performed on the log_2_ transformed concentration of CXCL10 (pg/ml) measured in culture supernatants: ${log}_{2}\left(Uninfected \left[CXCL10\right]\left(pg*{ml}^{-1}\right)\right)$ and ${log}_{2}\left(Infected \left[CXCL10\right]\left(pg*{ml}^{-1}\right)\right)$. For fold change of CXCL10, all analysis was performed on the log_2_ transformed value of infected CXCL10 divided by uninfected CXCL10 concentration: ${log}_{2}\left(\frac{Infected \left[CXCL10\right]\left(pg*{ml}^{-1}\right)}{Uninfected \left[CXCL10\right]\left(pg*{ml}^{-1}\right)}\right)$. Genome-wide association analysis for 3 CXCL10 phenotypes of interest was performed using PLINK v1.9^68^ with the QFAM-parents approach. The narrow sense SNP-based heritability (h^2^) was estimated using the method developed by Zaitlen et al*.*^46^.

### Linear modeling

Linear modeling analysis was performed in R version 3.6.0 using the lm function included in the base package^69^ for the 3 CXCL10 traits as follows (see R script in Supplementary Methods):

$${log}_{2}\left(Uninfected \left[CXCL10\right]\left(pg*{ml}^{-1}\right)\right)= rs2869462+Population+Cell \,Death$$

$${log}_{2}\left(Infected \left[CXCL10\right]\left(pg*{ml}^{-1}\right)\right)= rs2869462+Population+Cell\, Death+C.\,trachomatis\,burden$$

$${log}_{2}\left(\frac{Infected \left[CXCL10\right]\left(pg*{ml}^{-1}\right)}{Uninfected \left[CXCL10\right]\left(pg*{ml}^{-1}\right)}\right)= rs2869462+Population+Cell\,Death+ C.\,trachomatis\,burden$$

$${log}_{2}\left(\frac{Infected \left[RANTES\right]\left(pg*{ml}^{-1}\right)}{Uninfected \left[RANTES\right]\left(pg*{ml}^{-1}\right)}\right)= Population+Cell\,Death+ C.\,trachomatis\,burden$$

Variables are modeled in order as written, such that regression for each subsequent variable is calculated on the residual of the preceding variable(s). rs2869462 genotype was modeled as 3 categorical variables: CC, CG, or GG. Population was modeled as a categorical variable with 4 factors: ESN (Esan in Nigeria), GWD (Gambian in Western Divisions in the Gambia), KHV (Kinh in Ho Chi Minh City, Vietnam), and IBS (Iberian in Spain). Cell death was modeled as a continuous variable using the percent of dead cells as measured by 7AAD + staining by flow cytometry. *C. trachomatis* burden was modeled as a continuous variable as the percent of infected cells measured by flow cytometry of GFP produced by *C. trachomatis* (LGV-L2 Rif^R^ pGFP::SW2)-infected LCLs. All flow cytometry traits were measured using a Guava EasyCyte Plus flow cytometer (Millipore).

## Supplementary information

Supplementary Information 1.

Supplementary Information 2.

## Data Availability

The authors affirm that all data necessary for confirming the conclusions of this article are represented fully within the article, its tables and figures, and phenotype and GWAS datasets that are available for browsing and download at https://h2p2.oit.duke.edu.
