# Supplementary material for: Modeling of variables in cellular infection reveals CXCL10 levels are regulated by human genetic variation and the Chlamydia-encoded CPAF protease
Source: Sci Rep. 2020 Oct 26;10:18269. doi: 10.1038/s41598-020-75129-y (PMC7588472; doi:10.1038/s41598-020-75129-y)

Supplementary Information

Modeling of variables in cellular infection reveals CXCL10 levels are regulated by human genetic variation and the *Chlamydia*-encoded CPAF protease

Authors: Benjamin H. Schott[1†], Alejandro L. Antonia[1†], Liuyang Wang[1], Kelly J. Pittman[1], Barbara S. Sixt[1‡] Alyson B. Barnes[1], Raphael H. Valdivia[1], Dennis C. Ko[1,2,3*]

Affiliations

[1]Department of Molecular Genetics and Microbiology, School of Medicine, Duke University, Durham, NC 27710, USA

[2]Division of Infectious Diseases, Department of Medicine, School of Medicine, Duke University, Durham, NC 27710, USA

[3]Lead contact

[†]equal contribution

[‡]Current Address*:* Laboratory for Molecular Infection Medicine Sweden (MIMS), Umeå Centre for Microbial Research, Department of Molecular Biology, Umeå University, Umeå, Sweden

[*]To whom correspondence should be addressed: Dennis C. Ko, 0049 CARL Building Box 3053, 213 Research Drive, Durham, NC 27710. 919-684-5834. dennis.ko@duke.edu. @denniskoHiHOST

Supplementary Methods

R script used for modeling:

```
##############

master<-read.csv("PATH/TO/CXCL10_RANTES_Modeling.csv")


##RANTES Fold Change

glm.m1 = lm(master$log2.RANTES.Chlamydia...RANTES.Uninfected. ~
master$Population + master$X70hr.cell.death.Chlamydia +
master$Chlamydia.46hr..GFP.)


summary(glm.m1)


af <- anova(glm.m1)
afss <- af$"Sum Sq"
print(af)


print(afss)


print(cbind(af,PctExp=afss/sum(afss)*100))


##Remove the individual who is not genotyped at rs2869462 for modeling CXCL10
phenotypes
master<-master[-154,]
```

```
##CXCL10 Uninfected

glm.m1 = lm(master$log2.CXCL10.Uninfected. ~ master$rs2869462.Genotype +
master$Population + master$X70hr.cell.death.Uninfected)

summary(glm.m1)

af <- anova(glm.m1)
afss <- af$"Sum Sq"
print(af)

print(afss)

print(cbind(af,PctExp=afss/sum(afss)*100))

##CXCL10 Chlamydia
glm.m1 = lm(master$log2.CXCL10.Chlamydia. ~ master$rs2869462.Genotype +
master$Population + master$X70hr.cell.death.Chlamydia +
master$Chlamydia.46hr..GFP.)

summary(glm.m1)

af <- anova(glm.m1)
```

```
afss <- af$"Sum Sq"
print(af)

print(afss)

print(cbind(af,PctExp=afss/sum(afss)*100))

##CXCL10 Fold Change
glm.m1 = lm(master$log2.CXCL10.Chlamydia...CXCL10.Uninfected. ~
master$rs2869462.Genotype + master$Population +
master$X70hr.cell.death.Chlamydia + master$Chlamydia.46hr..GFP.)

summary(glm.m1)

af <- anova(glm.m1)
afss <- af$"Sum Sq"
print(af)

print(afss)

print(cbind(af,PctExp=afss/sum(afss)*100))

###########
```

Supplementary Table

Phenotype data for levels of CXCL10 and RANTES is provided in:

TableS1_CXCL10_RANTES_Modeling

Full gels

Figure 4C

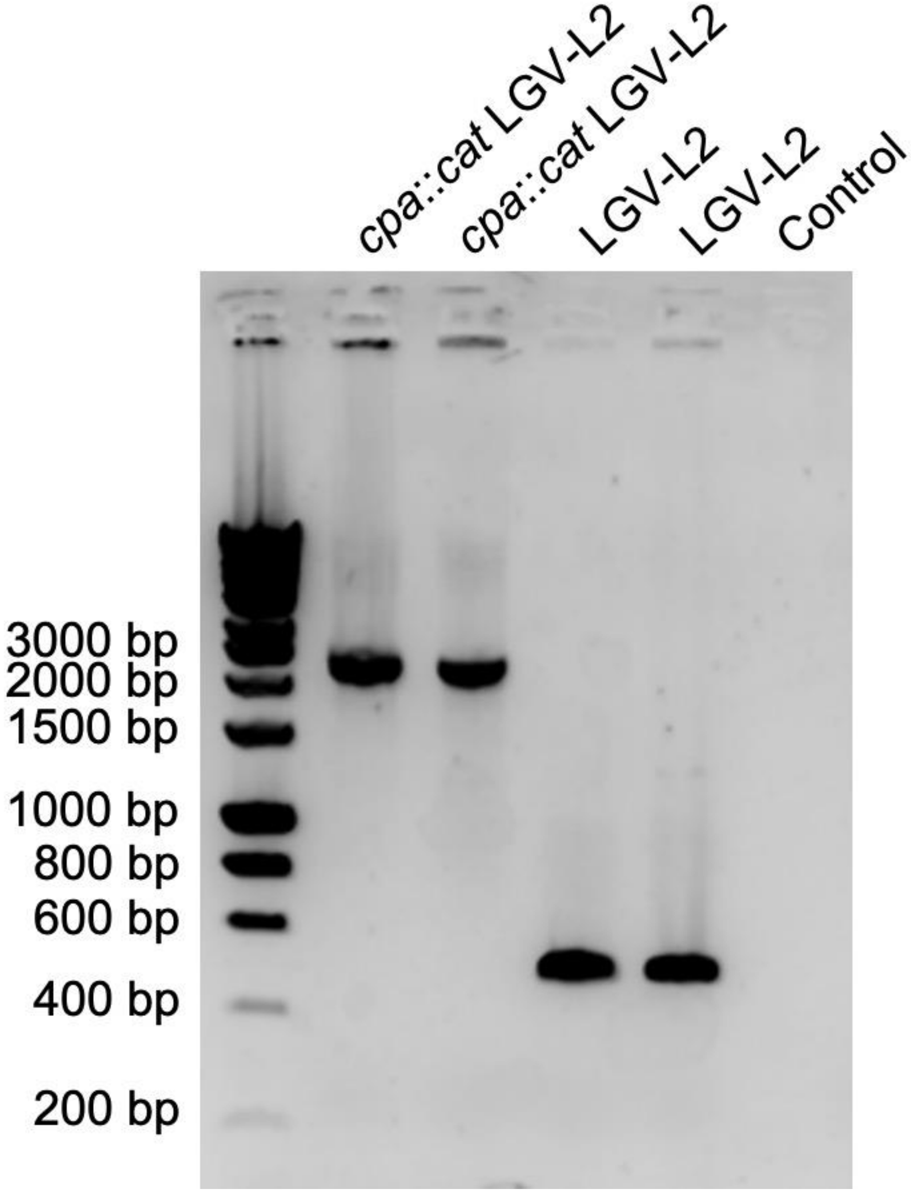

Supplement: Supplementary file 1 — Supplementary Information 1. [file 41598_2020_75129_MOESM1_ESM.pdf]
